# Pangenomes as a Resource to Accelerate Breeding of Under-Utilised Crop Species

**DOI:** 10.3390/ijms23052671

**Published:** 2022-02-28

**Authors:** Cassandria Geraldine Tay Fernandez, Benjamin John Nestor, Monica Furaste Danilevicz, Mitchell Gill, Jakob Petereit, Philipp Emanuel Bayer, Patrick Michael Finnegan, Jacqueline Batley, David Edwards

**Affiliations:** School of Biological Sciences, The University of Western Australia, Perth, WA 6009, Australia; cassandria.tayfernandez@research.uwa.edu.au (C.G.T.F.); benjamin.nestor@research.uwa.edu.au (B.J.N.); monica.danilevicz@research.uwa.edu.au (M.F.D.); mitchgill16@gmail.com (M.G.); jakob.petereit@uwa.edu.au (J.P.); philipp.bayer@uwa.edu.au (P.E.B.); patrick.finnegan@uwa.edu.au (P.M.F.); jacqueline.batley@uwa.edu.au (J.B.)

**Keywords:** pangenome assembly, graph pangenomes, presence absence variation, QTLs, trait discovery

## Abstract

Pangenomes are a rich resource to examine the genomic variation observed within a species or genera, supporting population genetics studies, with applications for the improvement of crop traits. Major crop species such as maize (*Zea mays*), rice (*Oryza sativa)*, *Brassica* (*Brassica* spp.), and soybean (*Glycine max*) have had pangenomes constructed and released, and this has led to the discovery of valuable genes associated with disease resistance and yield components. However, pangenome data are not available for many less prominent crop species that are currently under-utilised. Despite many under-utilised species being important food sources in regional populations, the scarcity of genomic data for these species hinders their improvement. Here, we assess several under-utilised crops and review the pangenome approaches that could be used to build resources for their improvement. Many of these under-utilised crops are cultivated in arid or semi-arid environments, suggesting that novel genes related to drought tolerance may be identified and used for introgression into related major crop species. In addition, we discuss how previously collected data could be used to enrich pangenome functional analysis in genome-wide association studies (GWAS) based on studies in major crops. Considering the technological advances in genome sequencing, pangenome references for under-utilised species are becoming more obtainable, offering the opportunity to identify novel genes related to agro-morphological traits in these species.

## 1. Introduction

Plant breeders have continually faced the challenge of increasing crop yield, nutrition, and disease resistance as the human population increases, and regions suitable for the production of crops shift with a changing global environment [1,2,3]. The construction of the first reference genome assembly for a crop species, rice (*Oryza sativa*) in 2002 [4], greatly improved the ability to associate traits with genomic regions, increasing the success of selection for specific traits that increase agronomically beneficial phenotypes. Improving genomic resources for crop species has predominantly focused on a limited number of high-yield, popular species such as wheat *(Triticum aestivum*) [5], rice (*Oryza sativa*) [6], maize (*Zea mays*) [7], barley (*Hordeum vulgare*) [8], soybean (*Glycine max*) [9,10], canola (*Brassica napus*) [11], and sorghum (*Sorghum bicolor*) [12]. These species are often referred to as major crops due to their extensive use in agriculture systems and high demand as food sources worldwide. The focus of genomic research and trait selection on major crops has led to many minor crops falling behind, limiting the opportunity to diversify the food bowl or discover the genetic basis for valuable traits within these species. Hence, under-utilised crops need investment to support their improvement and characterise traits that can potentially be transferred to major crops [13,14]. 

Reference genome sequences have recently been assembled for some under-utilised crop species such as yam bean (*Pachyrhizus erosus*) [15], kenaf (*Hibiscus cannabinus*) [16] and white fonio (*Digitaria exilis*) [17]. However, using a single reference leads to bias due to the significant structural variation (SV) observed within a species [18,19,20]. SVs can arise as a consequence of whole-genome duplication and subsequent fragmentation [21,22,23], or tandem and segmental duplication of genomic regions [24]. This duplication and fragmentation can lead to gene copy number variation (CNV) and gene presence/absence variation (PAV). CNV and PAV can also result from insertion of gene copies by transposable elements [25], de novo gene birth [22,26], introgression from closely related species, or horizontal gene transfer [27], which may affect heritable traits. Hence, single reference genomes do not reflect the gene content and diversity of a species, and improvements in the genomic resources over single reference genomes are needed in order to increase the success of genomics-based plant breeding for both major and under-utilised crop species.

Pangenomes are references that capture the genetic diversity of a species rather than a single individual and can reduce reference bias in genomic analysis, allowing more accurate prediction of traits [18,19]. A pangenome contains a core genome (shared among all individuals) and the variable or dispensable genome that is absent from one or more individuals [28]. The idea of a core and variable genome for a species represented by a pangenome was first described by Tettelin et al. in 2005 [28] and later proposed for use in plants by Morgante et al. in 2007 [29]. In 2014, the first plant pangenome was published, representing seven wild soybean (*Glycine soja*) individuals [30]. This was used to associate genes with the domestication traits of organ size, biomass, seed composition, flowering and maturity time, and disease resistance. Since then, other pangenomes have been constructed, including one representing 3 rice individuals [31], 10 *Brassica oleracea* individuals [32], 18 bread wheat individuals [20], 54 *Brachypodium distachyon* individuals [33], 53 canola individuals [34], 5 sesame (*Sesamum indicum*) individuals [35], 725 tomato (*Solanum lycopersicum*) individuals [36], 89 pigeon pea (*Cajanus cajan*) individuals [37], and 1961 cotton (*Gossypium* spp.) individuals [38] (Table 1). These provide valuable resources for understanding genetic variation in these species [39]. However, there are few pangenomic resources for under-utilised species, which limits the application of genomics to develop improved varieties of these crops. In this review, we examine several under-utilised crop species that lack pangenome resources and discuss the benefits the development of these resources may have for these species as well as the overall benefits to agriculture. The current methods for pangenome construction and trait analyses are also discussed. This review aims to provide a foundation for further studies to construct pangenomes for under-utilised crop species and improve their traits through plant breeding based on pangenomic analyses.

## 2. Under-Utilised Species

Many minor crops have yet to benefit from genomics-based breeding methods, despite many being important food sources in specific regions (Table 2). Under-utilised crop species cover a broad range of crop types, including cereal grains, vegetable, tubers, fruits, and crops with industrial uses (Table 3). Here, we describe several promising under-utilised crop species for each crop type and discuss the currently available genomic resources.

### 2.1. Cereal Grains

Wheat, maize, and rice constitute the major cereal grain crops and are responsible for supplying the majority of the global food requirement. However, these species are sensitive to drought and heat stress, leading to reduced yield or even crop failure in some environments [40]. Several under-utilised cereal crops are adapted to harsh environments and are alternatives to these major crops [41]. 

Little millet (*Panicum sumatrense*) is a small millet species native to India (hence its alternative name ‘Indian Millet’) and is primarily grown in semi-arid regions of Asia and Africa. This species requires minimal water and has a tolerance to drought and high salinity soil. However, Little millet is only grown in specific regions and few people consume it despite its nutritional benefits of high carbohydrate, dietary fibre, calcium, iron and Vitamin E content [42]. The genomic resources for Little millet are limited to the chloroplast genome sequence [43] and a transcriptome assembly [44] that has been used to characterise genes responsible for abiotic stress tolerance. This species lacks both a sequenced genome and a genetic map, limiting further study and genomics-based selection of traits.

White fonio (*Digitaria exilis*) is a panicoid grass and an under-utilised cereal crop from West Africa that is valued for its grain that is high in dietary fibre and protein [45]. The crop grows in hot, dry and low-fertility environments and requires no fertiliser or irrigation on poor-quality soils. However, white fonio has a low yield and minimal research has been undertaken into breeding to improve traits of this crop [46]. A genome sequence of white fonio has recently been assembled and annotated [17,47] and has been used for sequence-based genotyping [48]. Combining these genetic resources with other panicoid grass resources such as *Setaria italica* (foxtail millet) [49], *Cenchrus americanus* (pearl millet) [50], and *Panicum miliaceum* (proso millet) [51] through pangenomic and comparative genomic strategies may support white fonio research to benefit breeders and consumers [52].

### 2.2. Vegetable/Pulse Crops

The *Vigna* genus of legumes has many genetic resources, but few specifically for the under-utilised crop, moth bean (*Vigna aconitifolia*) [53]. Moth bean is a multipurpose legume that provides hot-season pasture and hay for livestock and seed. This species is the most heat-tolerant crop of the Asian *Vigna* species and is able to withstand drought conditions. Seeds and young pods of moth bean are suitable for human consumption and have a high vitamin and mineral content. While moth bean domestication is well studied and documented, the genetics of the domestication process is largely unknown. Genetic resources are largely limited to genetic linkage maps that can identify domestication-related traits and QTLs not present in moth bean, but that are present in other *Vigna* species [54]. These data can be integrated into genomic resources such as a pangenome, enhancing the genetic improvement of moth bean and related *Vigna* species. 

Lablab bean (Hyacinth bean, *Lablab purpureus*) is a leguminous crop that is commonly grown as a food source due to the seed having high protein content and a comparable nutritional profile to soybean [55]. In addition to being a source of nutrition, lablab bean is used to improve soil fertility as a cover crop and green manure [56]. Lablab bean has a higher drought tolerance compared to other commonly cultivated legumes and is able to grow across a wide range of climate and environmental conditions, withstanding temperatures from 18 °C to 50 °C and annual rainfalls from 200 to 2500 mm [57]. To enhance the production and benefits of lablab bean, new varieties need to be developed, especially those that are tailored to extended drought periods. Studies have largely focused on conventional breeding, but polygenic traits such as drought tolerance can be supported by more genomic research, which has been limited [58,59]. A draft genome for lablab bean was assembled in 2019 [60] and a chloroplast genome assembly in 2021 [61]. Further development of genomic resources through pangenomics would provide tools to help improve traits of this species and become an important safety net crop against the impact of climate change on legume production.

### 2.3. Tuberous Crops

The genus *Pachyrhizus* contains three yam bean species cultivated for their starchy tuberous root, *P. erosus*, *P. ahipa* and *P. tuberosus*. Yam bean is a regionally important crop in Mexico and Southeast Asia where it is eaten as part of many traditional dishes. Yam bean has a high yield and the crop can thrive in humid conditions [46,62]. The *P. erosus* tuber contains high vitamin C, iron, zinc and potassium [63]. Presently, there is a draft genome assembly *P. erosus* [15], and a flow cytometry study analysis [64], but *P. erosus* lacks the pangenome resources that would support studies of its abiotic stress traits for transfer to major legume crops [65]. 

African arrowroot (*Canna edulis*) is a tuber crop that originated in Central and South America and is distributed throughout Europe, North America and in tropical regions of the world. The tuber contains large amounts of starch which is highly viscous, often used in cakes, noodles, dye, and animal fodder [66]. African arrowroot is also known for its horticultural use in gardening and for the treatment of industrial wastewaters to remove pollutants such as nitrogenous and phosphorous compounds [67]. African arrowroot has a diverse germplasm and has over 1000 hybrids, making genomic studies into the species difficult. Presently, the only genomic resources for African arrowroot are a complete chloroplast genome [68]. Pangenome resources could be used to explore the diversity in gene content and compare genomic structures with related species. 

### 2.4. Industrial Crops

Kenaf (*Hibiscus cannabinus*) is an annual crop that is cultivated for the bast fibres that are produced on the stem bark of the plant. The species is the third most important source for fibre production after cotton and jute (*Corchorus* spp.) and it is often used in the production of paper, rope, building materials and as a livestock feed [69]. Kenaf has a high biomass yield and can acclimate to many different climates and soils [69], but little research has been undertaken on this species. A de novo transcriptome of kenaf was assembled in 2015 [69], and a mitochondrial genome sequence was assembled in 2018 [70]. These resources were recently supplemented in 2020 by a genome assembly, allowing for genes involved in the development of bast fibre and leaf shape to be identified [16]. Further study of the candidate genomic regions for bast fibre yield and quality-related traits using pangenomics could provide insights into yield and quality traits that could expedite the selection of elite traits.

Safflower (*Carthamus tinctorius*) is a thistle-like plant that is commercially cultivated for the vegetable oil extracted from its seeds. The species is found across Asia, Europe, Australia and the Americas [71], where it is popular due to the high content of linoleic acid and flavonoids, such as hydroxysafflor yellow A, in the oil [72]. Molecular studies have been undertaken in safflower primarily for fatty acid composition and flavonoid biosynthesis. Whole-genome sequencing efforts had been limited to short-read sequencing [71], but more recently, a chromosome-level reference genome assembly [73] was constructed that has allowed for evolutionary analysis of the divergence of safflower and the study of linoleic acid and flavonoid biosynthesis. While this whole-genome reference sequence has aided study into the genetic improvement of safflower, further improvement and understanding of how the Asteraceae family evolved and speciated can be achieved through the construction of pangenomic resources for safflower.

### 2.5. Fruits

Guava (*Psidium guajava*) is an important tropical and subtropical fruit of the Myrtaceae family, being the fourth most significant fruit crop in India [74]. The species is a highly sought-after export because it is a rich source of vitamin C, fibres and phytochemicals [75]. However, guava is vulnerable to the guava wilt pathogen *Nalanthamala psidii* and fruit flies, causing worldwide threats to the stability of guava production. Despite being economically valuable, there are few genomic resources for the species, especially resources that can be used to study the response of guava to biotic and abiotic stresses [76]. Most genomic resources for guava have only emerged in the early 2020s, including a genome assembly [76,77], high throughput and EST-based InDel/SNP markers [76] and a transcriptome assembly [78]. These resources lay the groundwork for improving the agronomic traits of guava by gene mapping and genomic selection that could be expedited through a pangenome.

Ethiopian banana (*Ensete ventricosum*) is a local crop that contributes to the food security of Ethiopia, providing a staple food source for approximately 20 million people [79]. The Ethiopian banana is an important dietary starch source and has uses in the production of fibre, medicine and other industrial products as well as an important role in stabilising soils, as well as being of cultural importance in Ethiopia [79]. Unlike most under-utilised crop species, pangenomics have been applied to Ethiopian banana with the species being included in a higher-level pangenome assembled for the Musaceae family [80]. This banana pangenome has allowed the identification of candidate regions for drought resistance, meristem initiation and stress resistance. The continued development of this pangenome will increase its value as a tool for trait improvement, broader diversity studies and evolutionary studies of banana species.

## 3. Developments in Pangenome Resources to Aid in the Breeding of Under-Utilised Crops 

**Figure 1:**
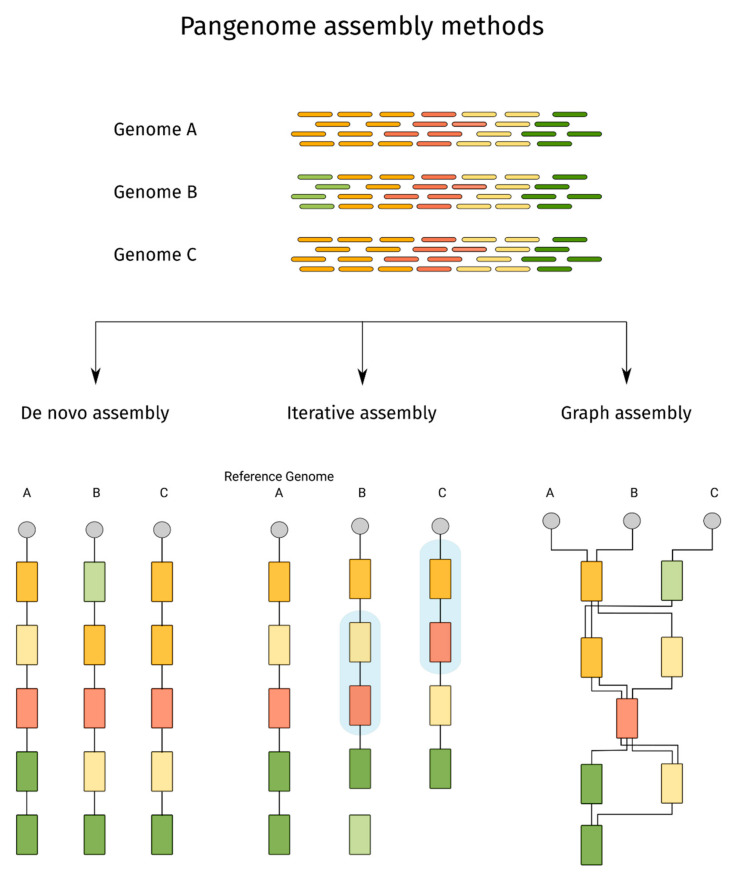
Scheme showing three pangenome assembly methods. Sequence reads from genomes A, B and C can be used to assemble the species pangenome using de novo method yielding three separate genomes that will be compared to define the core and variable regions. In the iterative assembly, genome A is assembled de novo and used as a reference for assembling the remaining genomes B and C. Because genome A has different genes from genome B and C, it may change the gene order in genome B (highlighted in the blue box) or collapsing CNV in genome C (highlighted in the blue box). In the iterative assembly, genes not represented in the reference genome (genome A) have to be assembled de novo and may lose their location information as shown by the green gene below genome B assembly. Graph pangenome assembly of genomes A, B and C represent the genes as interconnected nodes, each path representing a genome.

The three main approaches for pangenome construction used across genomic research are de novo assembly and comparison, iterative mapping and assembly, and graph-based assembly (Figure 1). The suitability of each approach depends on several factors, such as organism genome complexity, sequencing data quality and coverage, genetic similarity among individuals used for the pangenome construction and the intended final application of the pangenome. De novo assembly requires the individual genomes to be assembled separately, followed by whole genome comparison [29,30]. The iterative mapping and assembly approach involves mapping reads from different individuals to a starting reference genome, assembling the unmapped reads into novel contigs and then adding the novel contigs to the reference, forming a pangenome [32,34]. The iterative mapping approach and the de novo assembly approach are highly complementary, widely used and have been extensively discussed in other reviews [18,81,82]. 

Modelling suggests that as few as 10 representative individuals in a pangenome may capture the majority of gene diversity of a species. However, the advantage of increasing the number of individuals is that it permits an assessment of gene content variation across a population, and how this may change with breeding [9]. Recent pangenome studies of major crop species assess data from thousands or tens of thousands of individuals and include high quality chromosome-scale assemblies to further increase trait prediction accuracy [18,36]. 

Pangenome graphs are a relatively new pangenome construction method that combine the benefits of the iterative mapping and de novo assembly approaches. The method presents variation across multiple genomes as different paths along a graph of sequence or variant nodes. Pangenome graphs are constructed through whole-genome alignment, unassembled read alignment or de novo graph assembly [83,84]. Sequence graphs such as minigraph [85] represent nodes as short sequences, leading to highly complex networks that can present SVs in a manner where they can be compared among closely-related species [85,86]. Variation graphs, on the other hand, are a compact form of sequence graph used to present genetic variation across a population [87]. In variation graphs such as vg [88] or MGR [89], SNPs and SVs are represented by nodes and are connected when shared among individuals, allowing representation of large-scale SVs such as inversions and duplications [85,90,91]. 

Another type of pangenome graph is the practical haplotype graph (PHG) [92,93], which is a trellis graph representing genic and intergenic regions. PHGs avoid challenges in aligning repetitive and highly divergent regions through the use of a reference genome coordinate system that uses genes to anchor sequences [92,94], minimising errors due to reference bias, poor alignment and miscalled variants [95]. A common use of PHGs is to determine which haplotypes or genotypes of parental haplotypes that have been sequenced at high coverage are present in progeny that have been sequenced at low coverage. These graphs have been used in sorghum [92], maize [96] and cassava (*Manihot esculenta*) [95] to impute SNPs from low-coverage DNA sequence data. PHGs can support plant breeding as they can accurately capture the position of genomic variations among individuals. Advances in pangenomics are leading to the construction of higher-level pangenomes often referred to as super-pangenomes that represent genomic information at the genus level and above [80,97,98]. Super-pangenomes have the potential to aid introgression of traits from related species that can confer agronomic benefits. An example is alien introgression in *Brassica* breeding, where the Ogura fertility restorer gene system carried by the Rfo locus was introgressed into *B. napus* (which contains the *Brassica* A and C genomes) from closely related *Raphanus sativus* (radish) [99,100]. 

Super-pangenomes can support a more comprehensive view of gene PAV across species and provide a framework for evolutionary studies. The super-pangenome of banana identified gene differences between *Musa* and *Ensete* genera [80], as well as 12,310 new gene models in the species, forming distinct PAV clusters between the *Ensete* and *Musa* accessions. Variable genes related to flowering, meristem regulation and nutrient metabolism were enriched in the *Musa* accessions, reflecting the morphological diversity of *Musa* fruits [80]. Super-pangenomes at the genus level can also identify traits or genes lost during domestication or that have evolved in related species that can then be selected for in breeding. The latest soybean pangenome represented 1110 soybean individuals [10] and demonstrated that there had been a reduction in the number of protein-coding genes during domestication and subsequent breeding of elite varieties, with wild soybean having on average 620 more genes and a 21 Mbp larger genome than modern cultivars [10,101]. Studying how genes change in frequency between domesticated crops and their wild relatives using super-pangenomes can support the breeding of crops better adapted to diverse environments and more resilient to climate change.

Plant pangenome assemblies have shown that variable regions are often associated with biotic or abiotic stress [93], leading researchers to focus on the construction of pangenomes based on specific functional traits. These trait pangenomes aim to describe the landscape of genetic variation related to a trait. For example, resistance gene analogues (RGAs) have conserved domains and motifs that contribute to resistance to pathogens [102,103,104,105]. Thus, a pan-RGA can provide a platform to investigate the impact of genetic variation on plant resistance, as well as identify genetic markers for RGA profiling of species that may have limited genomic data [102]. A pan-RGA can be employed as a reference for resistance gene cloning [106,107]. In addition, trait pangenomes can be used to investigate the evolution and domestication of specific traits. For example, one study examined the differences in the nucleotide binding sites of leucine-rich repeat receptors (NLRs) during colonisation of new habitats by *Solanum chilense*, reinforcing that NLR evolution is constrained by their interaction with the products of other genes [108]. In the case of under-utilised species, trait pangenomes can help dissect the genetic variability associated with drought tolerance in the moth bean [109,110] and lablab bean [111,112], as well as potentially increase crop productivity by comparing yield-related genes with higher performing relatives. The functional analysis of the genetic diversity uncovered by pangenome studies is still largely unexplored but can be improved through the use of trait pangenomes, providing a foundation to accelerate breeding of under-utilised crop species that support food security globally.

## 4. The Breeding Potential of Under-Utilised Crop Species

Structural variation represented in pangenomes has been linked with pathogen resistance and tolerance to abiotic stress [32,113,114]. Identifying advantageous genes and alleles relies on associating pangenome SVs with phenotypic traits through genome-wide association studies (GWAS), quantitative trait loci (QTL) mapping or genomic selection [36,115,116]. As an example of pangenome-assisted GWAS analysis in major crops, a soybean graph-based pangenome with 29 assemblies identified a previously unknown PAV associated with seed luster [9]. Pangenome GWAS studies in other species detected 124 PAVs associated with yield and fibre quality in cotton [38], genes associated with seed traits and early leaf senescence in rice [6,117], PAVs associated with seed and flowering traits in canola [11], and 398 SNPs associated with agronomic traits in sorghum [12]. Pangenome GWAS and other functional comparisons support the linking of genomic variation with beneficial traits with an accuracy that linear single reference genomes are unable to provide. A functional pangenome analysis for under-utilised crops may uncover novel alleles related to agronomic traits in the variable genome that may be used for introgression into major crops or be used as genetic markers to improve traits of under-utilised crops. 

Characterising the relationship between SVs and differences in plant function requires integrating other data types, such as phenotype, metabolite and gene expression data, with the pangenome [82,118]. For example, SVs identified in a cotton pangenome with 890 accessions were compared through meta-GWAS and gene expression analysis to identify candidate genes related to yield and fibre quality. Genes identified include the previously uncharacterised gene GhIDD7 that was subsequently shown to control fibre length by using gene knockout with CRISPR-Cas9 [38]. Meta-GWAS was also employed in a soybean study using 17,556 accessions and associated phenotypic data to identify candidate genes related to agronomic traits, reporting several new loci, some of which were associated with multiple traits suggesting pleiotropic effects [119]. Leveraging previously published studies with biochemical analysis may help bridge the understanding of the effect of SVs on plant morphology. For example, although there are limited genomic resources for guava, a few studies have been conducted to investigate fruit and leaf metabolites [120,121] and fruit aroma volatiles of 27 guava accessions [122]. These datasets could be used to scan a guava pangenome for fruit related traits. A super-pangenome of yam bean species (*P. erosus*, *P. ahipa* and *P. tuberosus*) would provide a basis for integrating associated phenotype data. Multiple studies using agro-morphological traits collected for the yam bean varieties grown in Brazil, West Africa and Costa Rica have found significant variation between the genotypes employed in each study [123,124,125]. Integrating rich phenotype data with a yam bean super-pangenome could be used to infer the effects of SVs on phenotype, including traits directly related to plant performance such as day to flowering and maturity, plant height, and root biomass [125].

Previously identified genomic markers can be mapped to a pangenome reference to support the discovery of novel alleles. A recent pangenome study in tomato mapped 359 QTLs associated with volatile organic compounds [36,126]. These QTL regions were compared across diverse tomato populations, allowing the identification of alleles that can be used to improve fruit aroma [126]. Another study examined a tomato super-pangenome with 166 accessions from the wild ancestor *S. pimpinellifolium*, the semi-domesticated species *S. lycopersicum* var *cerasiforme*, and early domesticated *S. lycopersicum* var *lycopersicum*. They identified functional polymorphisms in the *LIN5*, *ALMT9*, *AAT1*, *CXE1*, and *LoxC* genes associated with fruit flavour. Beneficial haplotypes were identified that could be introgressed through conventional breeding [127]. These studies demonstrate the use of pangenomes to build on previous studies. 

Although there are limited genetic data for under-utilised crops, collating previous studies from closely related species may present encouraging results. For example, a study with finger millet (*Eleusine coracana*) used genotyping by sequence data to identify 109 SNPs, with five of these located in genes involved in flowering, maturity and grain yield [128]. Another study on finger millet identified 418 SNPs related to mineral micronutrient density that could be employed to improve grain nutrient quality [129]. Mapping previously reported markers onto a millet pangenome could improve our understanding of the genes related to agro-morphological traits in this under-utilised crop, thus supporting millet performance in the field. 

Advances in bioinformatics tools and data analysis will help accelerate under-utilised crop improvement using currently available genomic data. Machine Learning (ML) is a computational technology used to predict outcomes for specific problems based upon previous data. In bioinformatics, ML is becoming increasingly applied and optimised for crop-related advances in genomics and phenomics [118,130,131,132]. A recent study used random forest classification in conjunction with linkage disequilibrium mapping to identify pangenome PAV tags in domesticated barley with 83.6% accuracy, and in wild barley with 88.6% accuracy [133]. These barley PAV tags will help construct future barley pangenomes and can be applied to association analysis. Pangenomics ML has also been applied to understand gene loss mechanisms in *Brassica* [134]. It was demonstrated that gene loss was mainly associated with transposable elements in the diploid *B. oleracea* and *B. rapa,* while in the polyploid *B. napus,* the loss of genes was mostly associated with homoeologous recombination. ML can also be used for trait association in pangenomes, as seen in *B. napus*, where PAV associations were identified for disease resistance [135], and in pigeon pea for seed weight [37]. Here, using PAVs and SNPs from a pangenome rather than just SNPs derived from a single reference genome sequence as input when training ML models will increase the efficiency and reliability of prediction of traits in these crops. As the application of ML in crop science increases, these methods will become more common for the translation of pangenomic and crop trait data for under-utilised crop variety improvement.

## 5. The Future of Pangenomics in Breeding Under-Utilised Crops

Many of the advances in genomics and pangenomics have been driven by improvements in DNA sequencing technology. More accurate non-fragmented assemblies can now be generated using long-read sequencing methods such as Pacific Biosciences (PacBio) single-molecule real-time (SMRT) sequencing [136] or Oxford Nanopore Technologies (ONT) sequencing [137]. Long-read sequencing can now generate data with low error rates (between <1% and <5%, depending on the sequencer used) and span repetitive sequences, leading to pangenomes that contain fewer gaps and the ability to resolve placements of homeologous scaffolds [138,139]. Long-read sequencing also allows base modifications in complex repetitive regions to be analysed and for large SVs (>500 bp) to be assessed [140]. Improved sequencing and assembly methods have also allowed the capture of repetitive elements and complex inversions and translocations, allowing detection of SVs that would be missed in fragmented low-quality assemblies [81,141]. 

The additional SV data produced by these technologies can be translated to high-throughput and flexible molecular genetic markers for under-utilised crops. These markers can be used in breeding projects to maximise the efficiency of genomic selection for agronomically valuable traits [142]. However, the relatively high cost of generating long-read sequence data means that these high-throughput markers are not feasible for many genotyping applications. Furthermore, long-read sequencing has a large computational requirement in the analysis stage [143]. While software packages that analyse pangenomes and identify core and variable SNPs do exist, such as PanSeq [144], database systems for interpreting complicated SVs are rare. This rarity makes the use of long-read sequencing a challenge [145]. Nevertheless, the benefits of long-read sequencing for the construction of high-quality pangenomes makes it the approach of choice for future pangenomes, while the lower cost of short-read Illumina sequencing makes it more amenable for larger scale genotyping approaches.

**Figure 2:**
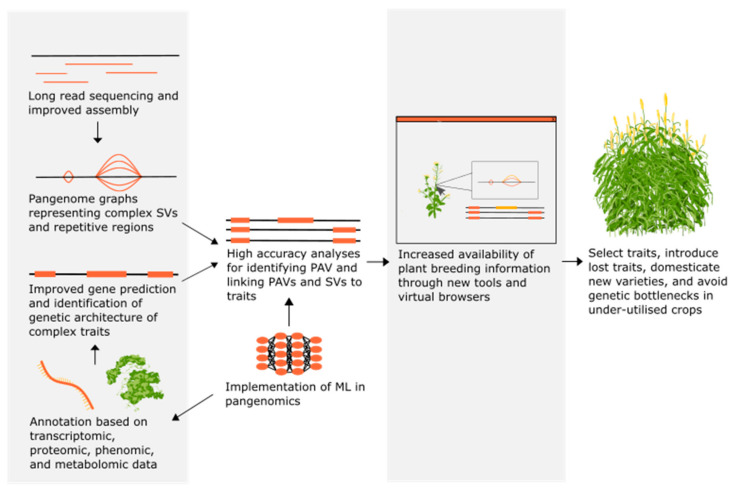
Predicted benefits to plant breeding from future developments in pangenomics. Improvements in pangenome assembly and annotation combined with machine learning (ML) technology will increase the accuracy of analyses on gene presence/absence variation (PAV) and structural variation (SV) in different individuals of crop species. These analyses will be available to plant breeders through new tools and browsers, allowing easier selection of traits and genetic diversity in crop plants.

As larger and more accurate genome assemblies are being produced, tools are being developed to annotate them more quickly and accurately [146]. Genome annotation tools such as BRAKER2 [147] and MAKER [148] combine ab initio (statistical model) and evidence-based gene predictions to produce higher quality annotations while still being relatively easy to use. However, annotation remains a bottleneck for large-scale genome and pangenome analysis, because gene prediction and functional annotation still lags behind assembly approaches [149,150,151]. In general, current gene prediction is complex. Most functional annotation tools draw from functional annotation databases that are either relatively small and manually curated, and therefore accurate, such as Swiss-Prot [152], or large and non-curated, and therefore potentially containing errors, such as the National Center for Biotechnology Information (NCBI) non-redundant database [153]. More accurate annotation methods are required to study differences in genetic architecture, because the detection of complex traits can be confounded when SVs and PAVs are incorrectly positioned. Future high-quality functional annotation will likely use transcriptomic, proteomic, phenomic, and metabolomic data with pangenomics together with approaches such as machine learning (ML) to increase accuracy. Currently, there are no universal ab initio methods or homology-based methods capable of aligning variations found in plant genomes with a reference pangenome [154]. To address this problem, research is underway to efficiently index, store and interrogate graphical representations of pangenomes that will lead to more accurate annotation [155] (Figure 2).

The full genetic potential of many under-utilised crops has yet to be fully realised, primarily due to a lack of resources that can be used to aid identification and selection of agronomically valuable traits. With the decreasing cost of sequencing, pangenomes for many under-utilised crop species can be assembled. These pangenomes can be used to identify genomic variation that can be studied with trait mapping tools such as GWAS and QTL, allowing the prediction of desirable crop traits using molecular markers [9,36]. By developing resources for under-utilised crops, novel genes related to agro-morphological traits can be detected and used to inform breeding programs or used for introgression into related major crop species. Furthermore, advancements in sequencing technologies will likely see pangenomes constructed with long-read DNA sequencing methods and chromosome-scale assemblies overtake single reference genomes for use in plant breeding research. The implementation of these pangenome assemblies in graph-based pangenomes and improvements in the accuracy of assembly and annotation tools will allow for more detailed analyses of the genetic constitution of under-utilised crops, and more efficient improvement of traits [88,92,131,156]. With pangenomes, existing genomic data and ML tools informing genetic breeding and gene editing, some of these climate-resilient and nutritious under-utilised crops show the potential to become alternative food sources or safety nets to major crops, supporting future increased agriculture system diversity and food security.

**Table 1:** Plant pangenomes constructed to date and method of assembly.

Species Name	# of Individual Genomes	Assembly Method	References
*Amborella trichopoda*	10	Iterative mapping and assembly	[76,77]
*Arabidopsis thaliana*	7	De novo assembly	[157]
*Brachypodium distachyon*	54	De novo assembly	[33,158]
*Brachypodium hybridum*	4	De novo assembly	[158]
*Brassica napus*	53	Iterative mapping and assembly	[34]
*Brassica napus*	8	De novo assembly	[11]
*Brassica oleracea*	10	Iterative mapping and assembly	[32]
*Cajanus cajan*	89	Iterative mapping and assembly	[37]
*Capsicum*	5	Iterative mapping and assembly	[156]
*Glycine max*	29	Graph-based de novo assembly	[9]
*Glycine max*	1110	Iterative mapping and assembly	[10]
*Gossypium*	1961	De novo assembly	[38]
*Hordeum vulgare*	20	De novo assembly	[8]
*Helianthus annuus*	287	De novo assembly	[159]
*Malus domestica*	91	De novo assembly	[160]
*Manihot esculenta*	57	Practical haplotype graphs	[161]
*Medicago truncatula*	15	De novo assembly	[162]
*Oryza sativa*	3	De novo assembly	[31]
*Oryza*	31	De novo assembly	[6]
*Poplar*	10	De novo assembly	[163]
*Sesamum indicum*	5	De novo assembly	[35]
*Solanum lycopersicum*	725	De novo assembly	[36]
*Sorghum bicolor*	398	Practical haplotype graphs	[92]
*Sorghum bicolor*	176	Iterative mapping and assembly	[12]
*Triticum aestivum*	18	Iterative mapping and assembly	[20]
*Zea mays*	4705	Practical haplotype graphs	[96]

**Table 2:** Research involving underutilised crops without genomic references.

Scientific Names	Common Names	Type of Resource	References
*Basella alba*	Malabar spinach	Reports of viruses infecting Malbar spinach	[164,165]
		Chromosome counts/Nuclear DNA quantification	[166]
*Calathea allouia*	Guinea arrowroot	Future prospects for underutilised medicinally valuable plants	[167]
*Couma utilis*	Milk tree	Identifying pollinators in edible Amazon fruit plants	[168]
*Crambe cordifolia*	Greater sea-kale	Ancestral chromosomal blocks in Brassiceae species	[169]
*Leopoldia comosa*	Tassel grape hyacinth	Identifying physiological responses	[170]
		Mineral content and chemical analysis	[171]
*Schinziophyton rautanenii*	Mongongo tree	Sustainability review	[172]
		Chemical composition of oil	[173]
*Ullucus tuberosus*	Ulluco	Viruses detected in ulluco	[174]
		High throughput sequencing to detect novel viruses in ulluco	[175]

**Table 3:** Underutilised crops with genetic resources.

Scientific Names	Common Names	Type of Genomic Resources	References
Cereal grains			
*Canna edulis*	African arrowroot	Chloroplast genome sequence	[68]
*Digitaria exilis*	White fonio	Genome assembly and annotation	[17,47]
		Genotype-by-sequencing and SNP data	[48]
*Panicum sumatrense*	Little Millet	Chloroplast genome sequences	[43]
		De novo transcriptome assembly	[44]
Vegetable/Pulse crops			
*Lablab purpureus*	Hyacinth bean/Lablab bean	Chloroplast genome assembly	[61]
		Draft genome assembly	[60]
		Upregulation of drought tolerant genes	[58]
		RFLP markers	[176]
*Solanum nigrum*	Black nightshade plant	Transcriptome sequence	[177,178]
		Chloroplast genome sequence	[179,180]
*Vigna aconitifolia*	Moth bean	Genetic linkage map	[54]
		Novel *Vigna* genetic resources	[53]
Tuberous crops			
*Pachyrhizus erosus*	Yam bean	Draft genome assembly	[15]
*Vigna vexillata*	Zombi pea or Wild cowpea	Anti-inflammatory bioactivity	[181]
		QTL analysis	[182]
		Molecular linkage analysis	[183]
		Hybridisation accession analysis	[184]
Industrial Crops			
*Carthamus tinctorius*	Safflowers	Transcriptome sequencing	[185,186]
		Chromosome-scale reference genome	[73]
		Chloroplast genome sequence	[187]
		Genetic mapping of SNPs	[71]
*Hibiscus cannabinus*	Kenaf	Mitochondrial genome assembly	[70]
		Genome assembly and annotation	[16]
		*De novo* transcriptome assembly	[69]
Fruit/Nuts			
*Bactris gasipaes*	Peach palm	Chloroplast DNA for phylogenetic study	[188]
		Macaúba palm transcriptome sequencing	[189]
		RNA-seq of tropical palms	[190]
		Plastome sequence	[191]
*Citrullus colocynthis*	Desert Watermelon or Wild watermelon	Gene markers	[192]
		Transcriptome assembly	[193]
		Genome Resequencing	[194]
*Elaeagnus angustifolia*	Russian olive or wild olive	Geographic study using machine learning	[195]
		Hi-C assembly	[196]
		Transciptome profiling	[197]
		Plant signalling regarding salt	[198]
*Ensete ventricosum*	Ethiopian Banana	Genome assembly	[199,200]
		Pangenome assembly	[80]
		Markers/Microsatellites	[201]
		Metabolite data	[202]
*Euterpe oleracea*	Açaí	Chemical genomic profiling	[203]
		Karyotype and genome size	[204]
*Psidium guajava*	Guava	Genome assembly	[76,77]
		Genome Markers	[76]
		RNA-seq/transcriptome assembly	[78]
*Vaccinium meridionale*	Agraz or Colombian Berry	Phylogenetic relationships within the *Vaccinieae* tribe	[205]
		Chemical, antimicrobial and molecular characterisation	[206]
		Characterisation of phenotypic plasticity	[207]
		Antiproliferative potential of Agraz juice	[208]

## Data Availability

Not applicable.
